# Protective Cellular Immunity Against Influenza Virus Induced by Plasmid Inoculation of Newborn Mice

**DOI:** 10.1155/1998/50472

**Published:** 1998

**Authors:** Adrian Bot, Simona Bot, Adolfo García-Sastre, Constantin Bona

**Affiliations:** 1 Department of Microbiology Mount Sinai School of Medicine New York USA; 2 Department of Biochemistry and Molecular Biology the Faculty of Medicine University of Salamanca Spain; 3 Center Molecular Biology and Genetics Kyoto University Japan; 4 Department of Microbiology Box 1124 Mount Sinai School of Medicine 1 Gustave L. Levy Place New York NY 10029 USA

**Keywords:** DNA immunization, newborns, cytotoxicity, influenza virus

## Abstract

Neonate organisms display an intrinsic disability to mount effective immune responses to infectious agents or conventional vaccines. Whereas low. doses of antigens trigger a suboptimal response, higher doses are frequently associated with tolerance induction. We investigated the ability of a plasmid-expressing nucleoprotein of influenza virus to prime a specific cellular immune response when administered to newborn mice. We found that persistent exposure to antigen following plasmid inoculation of neonates leads to a vigorous priming of specific CTLs rather than tolerance induction. The CTLs were cross-reactive against multiple strains of type A influenza viruses and produced IFN*γ* but no IL-4. The immunity triggered by plasmid inoculation of neonates was protective in terms of pulmonary virus clearance as well as survival rate following lethal challenge with influenza virus. Whereas the persistence of the plasmid at the site of injection was readily demonstrable in adult mice at 3 months after inoculation, mice immunized as newborns displayed no plasmid at 3 months and very little at 1 month after injection. Thus, DNA-based immunization of neonates may prove an effective and safe vaccination strategy for induction of cellular immunity against microbes that cause serious infectious diseases in the early period of life.


*Developmental Immunology*, 1998, Vol. 5, pp. 197–210
Reprints available directly from the publisher
Photocopying permitted by license only

© 1998 OPA (Overseas Publishers Association)
Amsterdam B.V. Published under license
under the Harwood Academic Publishers imprint,
part of the Gordon and Breach Publishing Group
Printed in Malaysia

### Protective Cellular Immunity Against Influenza Virus Induced by Plasmid Inoculation of Newborn Mice

ADRIAN BOT[a], SIMONA BOT[a], ADOLFO GARCÍA-SASTRE[b] and CONSTANTIN BONA[c*†]

[a]*Department of Microbiology, Mount Sinai School of Medicine, New York*; [b]*Department of Biochemistry and Molecular Biology of the Faculty of Medicine of the University of Salamanca, Spain*; [c]Center Molecular Biology and Genetics, Kyoto University, Japan

*(Received 4 March 1997; In final form 6 June 1997)*

Neonate organisms display an intrinsic disability to mount effective immune responses to infectious agents or conventional vaccines. Whereas low doses of antigens trigger a suboptimal response, higher doses are frequently associated with tolerance induction. We investigated the ability of a plasmid-expressing nucleoprotein of influenza virus to prime a specific cellular immune response when administered to newborn mice. We found that persistent exposure to antigen following plasmid inoculation of neonates leads to a vigorous priming of specific CTLs rather than tolerance induction. The CTLs were cross-reactive against multiple strains of type A influenza viruses and produced IFN$\gamma$ but no IL-4. The immunity triggered by plasmid inoculation of neonates was protective in terms of pulmonary virus clearance as well as survival rate following lethal challenge with influenza virus. Whereas the persistence of the plasmid at the site of injection was readily demonstrable in adult mice at 3 months after inoculation, mice immunized as newborns displayed no plasmid at 3 months and very little at 1 month after injection. Thus, DNA-based immunization of neonates may prove an effective and safe vaccination strategy for induction of cellular immunity against microbes that cause serious infectious diseases in the early period of life.

*Keywords:* DNA immunization, newborns, cytotoxicity, influenza virus

### INTRODUCTION

Various vaccine strategies were shown to induce a cytotoxic immune response in adult organisms. An effective induction of CTL immunity depends on the ability of the vaccine to direct the epitopes to the MHC-class I pathway of presentation (Townsend et al., 1985). Live-attenuated recombinant vectors (Aggarwal et al., 1990; Tartaglia et al., 1992), peptide or protein formulations in liposomes (Harding et al., 1991; Nair et al., 1992), lipid-tailed peptides (Allsopp et al., 1996), or recombinant Ty viruslike particles (Griffiths et al., 1991) were shown to induce specific CTL responses. DNA immunization emerged as a

*Corresponding author.
†Present address: Department of Microbiology, Box 1124, Mount Sinai School of Medicine, 1 Gustave L. Levy Place, New York, NY 10029.

promising strategy of vaccination tested in numerous experimental systems and able to trigger in adult organisms long-term cellular (Ulmer et al., 1993; Yankauckas et al., 1993) and humoral (Davis et al., 1993; Robinson et al., 1993) immunity with protective ability in some cases (Whalen, 1996). DNA-based vaccination offers some advantages over the other vaccine strategies: ability to induce a CTL immune response in absence of vector replication and strong adjuvants, long duration of memory presumably due to the persistence of the antigen, and a relatively easy and rapid manufacture procedure that lowers the fabrication costs (Whalen, 1996).

Compared with adults, young organisms display particular immune responsiveness to antigens. Early studies showed that newborns are very susceptible to tolerance induction for alloantigens (Billingham et al., 1956) and polysaccharides (Bona et al., 1978) leading to the paradigm of neonatal tolerance as a mechanism for self-/nonself-discrimination. More recent studies however, showed that newborns are able to mount immune responses, although qualitatively different as compared with adult organisms. Thus, both *in vitro* and *in vivo* experiments demonstrated that newborn lymphocytes have an intrinsic tendency to respond via a Th2 pattern to polyclonal stimulators (Adkins et al., 1993) or antigens (Barrios et al., 1996), respectively. Further, high-zone tolerance in neonates was associated with a strong Th2 type of immune response (Forsthuber et al., 1996; Sarzotti et al., 1996), suggesting that active suppressive mechanisms rather than precursor deletion may be responsible for neonatal low responsiveness to foreign antigens. Recent studies showed that targeting the antigen to professional antigen-presenting cells (APCs) (Ridge et al., 1996), use of certain adjuvants (Forsthuber et al., 1996), and decreasing the dose of immunogen (Sarzotti et al., 1996) were followed by induction of significant cellular immune responses in neonates. Thus, at least in some circumstances, immunization of newborns triggers Th1 and CTL immune responses. In light of these recent studies, the particular immune responsiveness of newborns consisting in tolerance susceptibility and Th2-biased immune responses seems to be due to predominant presentation of antigen by nonprofessional APC rather than to intrinsic characteristics of neonatal T lymphocytes.

We investigated the ability of the NPV1 plasmid-expressing nucleoprotein (NP) of influenza virus strain A/PR8/34 (PR8) to induce cytotoxic immune responses following i.m. inoculation of newborn BALB/c mice. Previous studies showed that injection of NPV1 plasmid in adult mice primed a strong CTL response that protected the animals after challenge with a heterologous strain of influenza virus (Ulmer et al., 1993). It is known that whereas hemagglutinin (HA) encodes the major B and Th epitopes responsible for the generation of protective antibodies (Virelizier, 1975), NP and other inner proteins encode CTL epitopes that participate to the clearance of virus (Yap et al., 1978; Lin and Askonas, 1981; Lukacher et al., 1984). It was shown that anti-NP antibodies do not mediate any protective effects (Epstein et al., 1993). Thus, we asked the question if persistent antigen exposure following DNA immunization of newborn mice primes a protective cellular immune response or, alternatively, induces specific immunological tolerance.

### RESULTS

### CTL Priming by Neonatal Immunization with NPV1 Plasmid

To determine the response of BALB/c mice immunized as newborns with NPV1 plasmid, we studied the primary and secondary cytotoxicity of splenocytes from animals sacrificed at ages of 1 (Figures 1A and 1B) and 3 months (Figures 1C and 1D), respectively. Half of the animals were boosted with PR8 live virus i.p. 1 week previous to sacrification, in order to assess the responsiveness of neonates immunized with NPV1 plasmid to influenza virus. We carried out in parallel, analogous experiments in adult BALB/c mice and we included several controls in both age groups. As shown in Figure 1A, both neonates and adult mice immunized with NPV1 and boosted with live PR8 virus displayed significant primary cytotoxicity for the NP 147-155 peptide, the major CTL epitope of NP in the H-$2^d$ haplotype. By contrast, neither the mice injected with control plasmid (CP) and boosted with PR8 virus 1 week prior to sacrification (Figure 1A) nor the neonates immunized at birth with 5 $\mu$g PR8 virus and boosted with virus 3 weeks later (data not shown) displayed primary cytotoxicity against the same peptide. After *in vitro* expansion with PR8-infected BALB/c splenocytes, adult and newborn mice immunized with NPV1 plasmid or PR8 virus displayed significant secondary cytotoxicity for NP 147-155 peptide (Figure 1B). Mice immunized with NPV1 plasmid and boosted with PR8 virus consistently exhibited the highest specific lysis values. No primary or secondary CTL activity was observed in the case of animals injected only with CP, which lacks the NP open-reading frame (Figure 1A). These results indicate that NPV1 immunization had a significant priming effect on the generation of NP-specific CTLs, both in neonate and adult BALB/c mice. Similar results were obtained in animals studied 3 months after the completion of immunization with NPV1 (Figures 1C and 1D), showing that CTL immunity primed by NPV1 injection of neonates persists at least 3 months.

To estimate more precisely the priming effect of NPV1 injection in neonates and adult mice, we carried out CTL precursor (pCTL) frequency estimation by limiting dilution analysis using splenocytes from animals immunized with plasmid or PR8 virus, or immunized with NPV1 and boosted with PR8 virus. As shown in Figure 2, mice immunized as neonates or adults with NPV1 and boosted with PR8 virus displayed higher frequencies of PR8 virus-

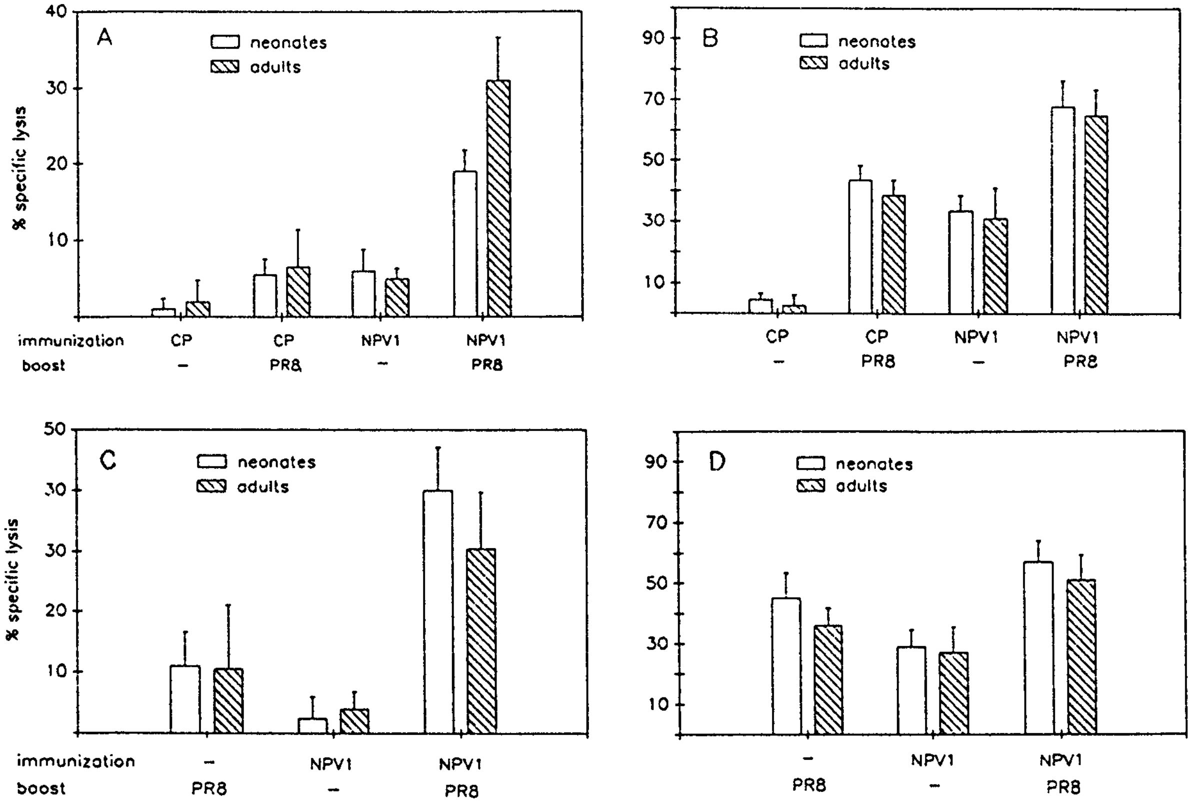

FIGURE 1 Primary (A and C) and secondary (B and D) cytotoxic activity of splenocytes from mice immunized 1 month (A and B) or 3 months (C and D) previously with NPV1 plasmid. Mice were immunized, as described in Materials and Methods, and sacrificed after 4 or 12 weeks since the last inoculation with plasmid or 1 week after i.p. inoculation of PR8 virus, respectively. For the secondary CTL assay, responder cells were stimulated with PR8 virus. Target cells were NP 147-155 peptide-coated or uncoated P815 cells. The results at E:T = 160:1 in the case of primary cytotoxicity (A and C) and E:T = 100:1 for the secondary cytotoxicity (B and D) are shown as a mean of percent specific lysis ±SD in groups of three mice. Results at lower E:T ratios are consistent with those shown in the figure.

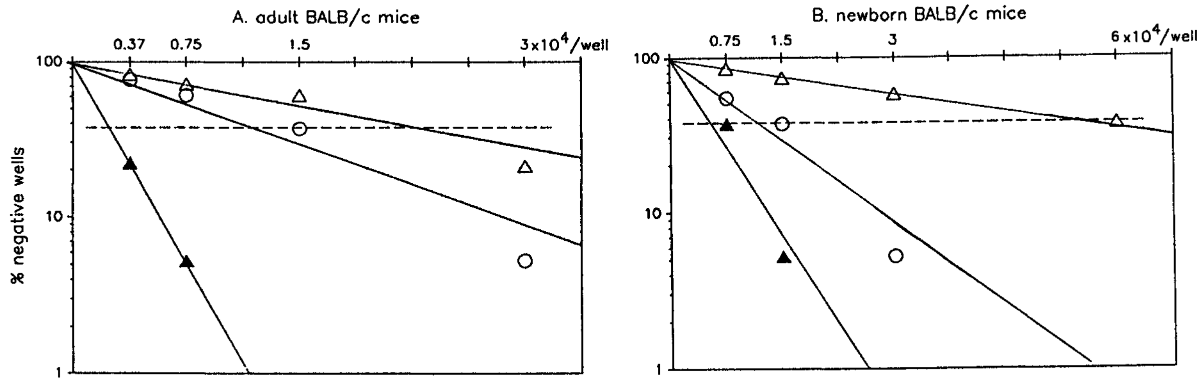

| immunization | frequency | 1/frequency | relative frequency |
|---|---|---|---|
| ○ PR8 | $8.3x10^{-5}$ | $1.2x10^{4}$ | 1 |
| △ NPV1 | $4.3x10^{-5}$ | $2.3x10^{4}$ | 0.5 |
| ▲ NPV1 and PR8 | $4.0x10^{-4}$ | $2.5x10^{3}$ | 4.8 |

| immunization | frequency | 1/frequency | relative frequency |
|---|---|---|---|
| ○ PR8 | $8.3x10^{-5}$ | $1.2x10^{4}$ | 1 |
| △ NPV1 | $1.9x10^{-5}$ | $5.4x10^{4}$ | 0.2 |
| ▲ NPV1 and PR8 | $2.0x10^{-4}$ | $5.0x10^{3}$ | 2.4 |

FIGURE 2 Frequency of PR8-virus specific pCTLs in spleens of mice immunized as adults (A) or neonates (B) with NPV1 plasmid. The immunization with NPV1 was completed 4 weeks before analysis. Some mice were boosted with live PR8 virus 7 days previous to sacrification. Responder cells pooled from three mice in each group were cultured in 24 replicates at each titer before 5-day stimulation with irradiated BALB/c splenocytes infected with PR8 virus. Microcyto toxic assays were carried out against PR8-infected or noninfected P815 target cells. 1/pCTL frequency was estimated after linear regression ($-1.0 < r < -0.95$ in all cases). Relative frequency of pCTLs was estimated against the pCTL frequency in spleens of PR8-immunized BALB/c mice.

specific pCTL in the spleen as compared with animals immunized with PR8 or NPV1 alone. Immunization with NPV1 primed two to five times less pCTLs as compared with the live virus. Notably, the efficacy of immunization with NPV1 was approximately two times higher in adult mice versus neonates, as shown by a comparison of the relative frequency data (Figure 2). Thus, neonatal injection of NPV1 was followed by the priming of virus-specific CTLs rather than tolerance induction.

### IFN$\gamma$ Production by T Cells Primed Subsequent to Neonatal Immunization with NPV1

As an independent means to assess the priming of virus-specific T cells following the neonatal injection of plasmid, we measured the production of IFN$\gamma$ by T splenocytes. One month after the completion of immunization with NPV1, T cells were harvested and restimulated *in vitro* with NP 147-155 peptide in presence of APCs. Estimation of IFN$\gamma$ concentration in the supernatants showed significantly higher values in the case of mice immunized with NPV1 and boosted with PR8 virus, compared to mice immunized with PR8 virus alone (Figure 3A). No significant IFN$\gamma$ production was noted in the case of non-fractionated T cells from mice immunized as newborns with NPV1 alone. Enrichment of $CD8^+$ T cells by negative selection previous to *in vitro* restimulation revealed significant concentrations of IFN$\gamma$ produced by cells from mice immunized as neonates with NPV1 alone (Figure 3B). No IL-4 was detected in culture supernatants following restimulation of T cells with NP 147-155 peptide, irrespective of the immunization protocol (data not shown). Together, these data show that NPV1 inoculation of newborn mice primed NP 147-155-specific $CD8^+$ T cells with the ability to produce IFN$\gamma$ and to expand subsequent to the virus restimulation.

### Cross-Reactivity of CTLs Primed by Neonatal Immunization with NPV1

We further investigated the cross-reactive pattern of CTLs induced by NPV1 immunization of newborn mice by performing secondary cytotoxic assays after *in vitro* expansion with PR8 infected stimulator cells. As target cells, we used P815 cells infected with A/

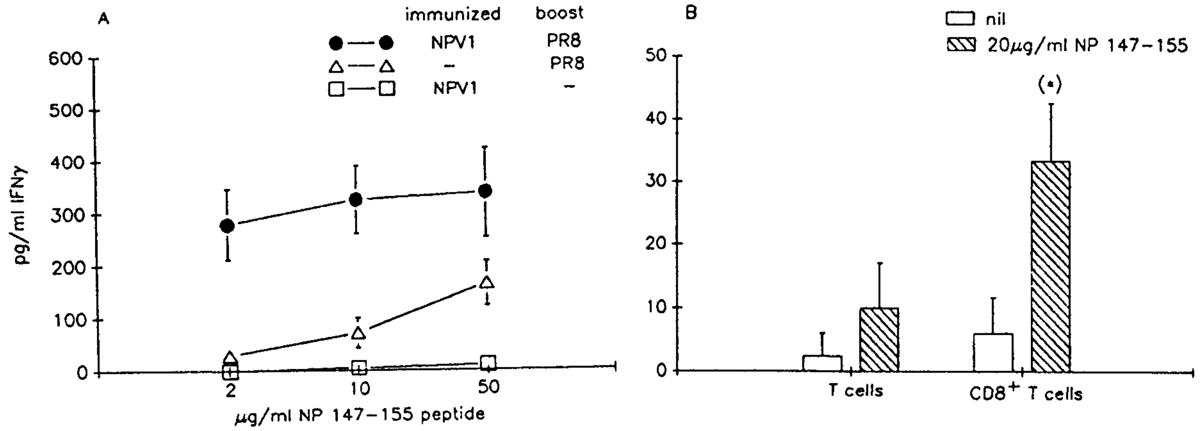

FIGURE 3 IFNγ production of T cells from mice immunized as newborns with NPV1. Nonfractionated T cells from newborn mice nonimmunized or immunized with NPV1 and boosted or not with PR8 virus were incubated with APCs in presence of various concentrations of NP 147-155 peptide (A). In a different experiment (B), nonfractionated or CD4-depleted T cells from mice immunized as newborns with NPV1 were restimulated in vitro with NP 147-155 peptide. In all cases, T cells were pooled from three mice in each group. IFNγ concentration in supernatants was measured by ELISA and results were expressed as mean of triplicates ±SD. (*) p of the t test < 0.05.

PR8/34, A/HK/68, A/Japan/57, or B/Lee/40, respectively. It was previously established that whereas NP of influenza viruses type A is relatively conserved even among various subtypes, major differences occur in HA (reviewed in Krug, 1989). As shown in Figure 4, splenocytes from mice immunized with NPV1, PR8 virus or immunized with NPV1 plasmid and boosted with PR8 virus displayed significant CTL activities against type A influenza viruses but not against B/Lee virus, which is a type B influenza virus. Again, mice immunized with NPV1 and boosted with PR8 virus showed the highest secondary cytotoxic activities, confirming the priming ability of NPV1 in neonates (Figure 4B). Concordant with previous studies showing that NPV1 induces type A cross-reactive CTLs in adult mice (Ulmer et al., 1993), these results demonstrate the same cross-reactive ability of CTLs primed by NPV1 injection of newborns.

### Pulmonary Virus Titers in Mice Immunized with NPV1 as Neonates and Challenged with Lethal Doses of Influenza Viruses

The protective ability of immunity induced by NPV1 in neonates and adult mice was investigated following aerosol infection with one $LD_{100}$ of PR8 or HK virus 1 and 3 months after completion of immunization. Pulmonary virus titers were estimated in groups of three mice at day 3, 7, and 16 after infection. As shown in Table 1, whereas PR8-immunized adult mice displayed no virus on day 3 after homologous

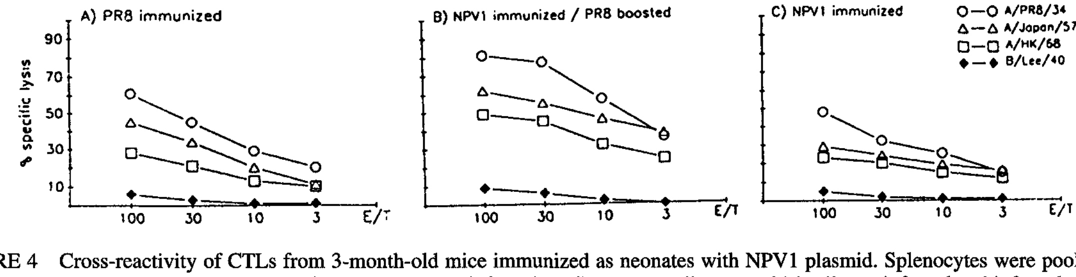

FIGURE 4 Cross-reactivity of CTLs from 3-month-old mice immunized as neonates with NPV1 plasmid. Splenocytes were pooled from three animals in each group and expanded in vitro with PR8-infected APCs. Target cells were P815 cells noninfected and infected with type A viruses (open simbols) or B/Lee virus. Results were expressed as percent-specific lysis values for various E:T ratios in the case of PR8-immunized mice (A), NPV1 immunized and PR8 boosted (B), or NPV1 immunized mice (C).

TABLE I Effect of Immunization with NPV1 Plasmid on Pulmonary Virus Titer after Lethal Aerosol Challenge with PR8 or HK Influenza Viruses

| Animal groups | Immunization | Pulmonary virus titer[a] | | |
|---|---|---|---|---|
| | | Day 3[b] | Day 7 | Day 16 |
| **Challenge with PR8** | | | | |
| Adult | Nil | 4.6 ± 0.5 | 3.8 ± 0.1 | +[d] |
| 1 month after immunization | PR8 virus[c] | 0 | 0 | ND[e] |
| | Control plasmid | 4.8 ± 0.1 | 3.7 ± 0.5 | + |
| | NPV1 plasmid | 4.0 ± 0.3 | 0.9 ± 1.5 | 0 |
| Adult 3 months after immunization | NPV1 plasmid | 4.8 ± 0.1 | 0.2 ± 0.2 | 0 |
| Newborn | Control plasmid | 5.9 ± 0.0 | 4.6 ± 0.2 | + |
| 1 month after immunization | NPV1 plasmid | 4.5 ± 1.2 | 1.2 ± 2.1 | 0 |
| Newborn 3 months after immunization | NPV1 plasmid | 4.1 ± 0.5 | 0.9 ± 1.2 | 0 |
| **Challenge with HK** | | | | |
| Adult | Nil | 6.4 ± 0.7 | 5.7 ± 0.3 | + |
| 1 month after immunization | PR8 virus | 5.7 ± 0.3 | 0 | ND |
| | Control plasmid | 6.8 ± 0.1 | 5.7 | + |
| | NPV1 plasmid | 5.8 ± 0.1 | 0.6 ± 1.1 | 0 |
| Adult 3 months after immunization | NPV1 plasmid | 4.3 ± 0.4 | 0 | 0 |
| Newborn | Control plasmid | ND | ND | ND |
| 1 month after immunization | NPV1 plasmid | 6.6 ± 0.3 | 5.1 ± 0.6 | + |
| Newborn 3 months after immunization | NPV1 plasmid | 5.6 ± 0.2 | 0 | 0 |

[a]Pulmonary virus titer was estimated by chicken red blood cell hemagglutination after 48 hr incubation of serial dilutions of tissue homogenates with MDCK cells. The results are expressed as mean ±SD of $\log_{10}$ $TCID_{50}$ measured individually for each animal in groups containing three mice.
[b]Mice were challenged with one $LD_{100}$ aerosolized PR8 virus ($1.5 \times 10^4$ $TCID_{50}$) or HK virus ($1.5 \times 10^5$ $TCID_{50}$) at day 0 and three mice were sacrificed at day 3 and 7 after infection. All survivor mice were sacrificed at day 16 and pulmonary lung titers were measured.
[c]Mice were immunized i.p. with live PR8 virus 7 days previous to sacrification.
[d]No surviving mice at day 16 after challenge.
[e]Measurement of pulmonary virus titer was not carried out.

infection, significant pulmonary virus titers were noted on day 3 following the challenge with HK virus. Adult mice immunized with PR8 and challenged with HK virus cleared the pulmonary virus between day 3 and 7 after infection. In contrast, mice noninjected or injected with CP failed to clear the virus and did not survive until day 16 following the challenge. In terms of virus lung titers, no significant differences were noted between mice immunized with NPV1 and nonimmunized mice on day 3 following homologous or heterologous challenge. In contrast, at day 7 following PR8 virus infection, significant decrease of pulmonary virus titer was noted in case of adult and newborn mice immunized with NPV1 at 1 or at 3 months after completion of immunization (in all cases, $p$ of $t$ test $< 0.05$). All mice immunized with NPV1 that survived until day 16 after infection completely cleared the virus from their lungs. Similar results were noted following heterologous challenge, except the group of mice immunized as newborns and challenged 1 month later that failed to clear the virus. Interestingly, when infected at the age of 3 months, mice immunized with NPV1 as neonates and challenged with HK virus cleared the virus by day 7. Thus, the kinetics of virus reduction in lungs of mice immunized with NPV1 as adults or neonates is consistent with a protective role for T-cell immunity, in particular virus-specific CTLs.

### Survival of Mice Immunized as Neonates with NPV1 and Challenged with Lethal Doses of Influenza Viruses

We also followed the survival of NPV1-immunized adult and neonates after challenge with one $LD_{100}$ of PR8 or HK virus (Figure 5). Adult mice immunized with NPV1 and challenged 1 or 3 months later with PR8 virus displayed a statistically significant survival rate ($p$ of Fisher's test $< 0.05$) compared with control mice nonimmunized or injected with CP (Figures 5A, 5B, and 5C). Although PR8 immunization conferred 100% protectivity, the percentage of survivors was lower in the case of NPV1-immunized mice, underlining the role of virus-specific protective antibodies. Newborns immunized with NPV1 displayed no significant survival 1 month later after lethal challenge with PR8 virus ($p > 0.10$; Figure 5B). In contrast, 3-month-old mice immunized as neonates with NPV1 plasmid showed a statistically significant survival rate after infection with the same dose of PR8 virus, comparable to that noted in adult mice immunized with NPV1 plasmid ($p < 0.05$; Figure 5C). Lower survival rates were noted in mice immunized as neonates or adults with NPV1 and challenged with one $LD_{100}$ of HK virus (Figures 5E and 5F). Again, slightly higher protection was evident in mice challenged 3 months after the completion of immunization.

### Local Recruitment of Effector Cells in Mice Immunized as Newborns with NPV1 and Challenged with PR8 Virus

In order to better characterize the protective mechanisms triggered by NPV1 immunization of newborn mice, we estimated the number of PR8-specific pCTLs in the lungs of 3-month-old mice following aerosol challenge. Compared with nonimmunized age-matched BALB/c mice, 3-month-old mice immunized as neonates with NPV1 plasmid displayed increased numbers of specific pCTL in lungs and spleens (Table 2). On a ratio basis, the difference was more important in lungs at day 3 after infection, suggesting that an early local recruitment of specific CTLs plays important roles in effective recovery from viral pneumonia and limitation of DTH lesions.

### Persistence of the Plasmid at the Site of Injection

We studied the presence and persistence of NPV1 plasmid in gluteal muscle and anterior tibial muscle of mice immunized as newborns and adults, respectively. Surprisingly, whereas the plasmid was easily detectable at the site of injection in adult mice 1 month after immunization, neonates immunized with NPV1 displayed weak PCR signals at the age of 1 month (Figure 6). At 3 months after the completion of immunization, some adult mice still presented detectable plasmid at the site of injection. In contrast, plasmid was detected in none of the mice immunized as newborns and studied 3 months later (Figure 6). These results suggest that the persistence of the plasmid at the injection site is more limited in mice injected as neonates. Whereas the plasmid persisted more than 3 months in some mice immunized as adults, it persisted less than that in all of the mice immunized as newborns.

### DISCUSSION

Injection of newborn mice with NPV1 led to the priming of CTLs with the ability to lyse virus-infected cells and to secrete IFN$\gamma$ following restimulation with NP 147-155 peptide, which is recognized in context of MHC-I molecules. The primary cytotoxic assay results and the estimation of virus-specific pCTL frequency in spleens of mice immunized with NPV1 and boosted with virus indicated that CTLs primed during the early period of life have the ability to proliferate on subsequent antigen exposure. This conclusion was confirmed by *in vitro* proliferation of T cells from mice immunized as newborns with NPV1, following restimulation with PR8 virus or NP 147-155 peptide (data not shown). Furthermore, CTLs primed by neonatal immunization with plasmid were promptly recruited into the lungs of mice infected with virus and contributed to the clearance of virus and the increased survival following lethal

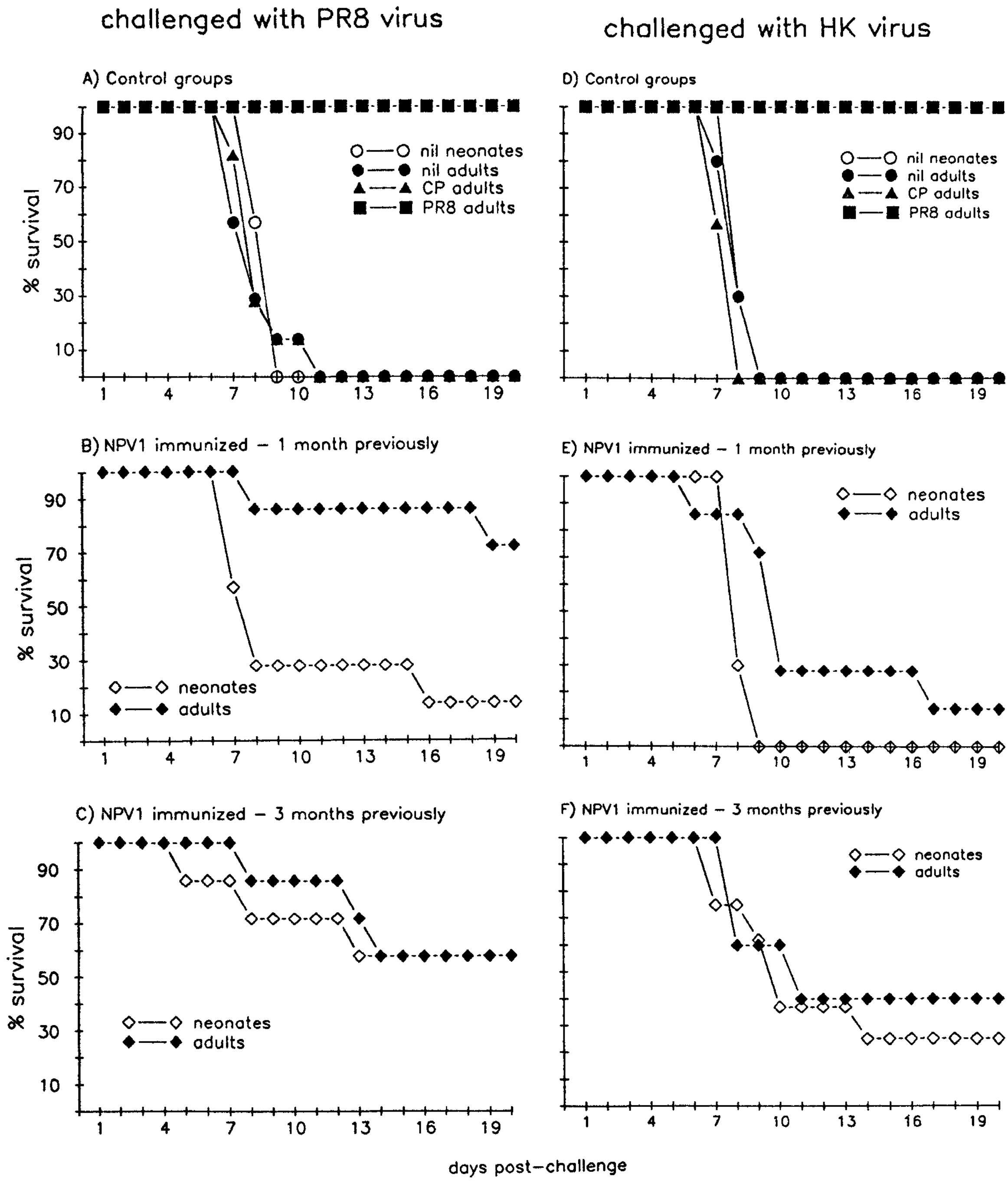

FIGURE 5 Survival of NPV1 plasmid-immunized mice challenged with lethal doses of PR8 or HK viruses. Control animals: Nonimmunized neonates, nonimmunized adults, and CP-injected or PR8-immunized adults are shown in panels A and D. Adult (closed symbols) or newborn (open symbols) mice were immunized with NPV1 and infected with a dose of $1.5 \times 10^4$ $TCID_{50}$ PR8 virus 1 (B) or 3 (C) months after the last plasmid inoculation. In parallel experiments, mice were infected with $1.5 \times 10^5$ $TCID_{50}$ of HK virus at 1 (E) or 3 (F) months after the completion of immunization. Each group was composed of five to ten animals and the percent of survivors was represented against time since challenge. Mice that survived until day 20 recovered their original weight and displayed no infectious virus in the lungs.

TABLE II Frequency of CTL Precursors in Lungs and Spleens of 3-Month-old Mice Challenged with a Lethal dose of Influenza Virus

| Group | Day post | Spleen | Lung | | |
|---|---|---|---|---|---|
| | challenge | 1/Frequency[a] | Total[b] | 1/Frequency[a] | Total[b] |
| BALB/c immunized with NPV1[c] | 3 | $1.3 \times 10^5$ | $10^3$ | $6.3 \times 10^4$ | $2 \times 10^2$ |
| | 7 | $2.4 \times 10^4$ | $4.2 \times 10^3$ | $5.2 \times 10^3$ | $2 \times 10^3$ |
| BALB/c nonimmunized | 3 | $6.6 \times 10^5$ | $10^2$ | $5.5 \times 10^5$ | 10 |
| | 7 | $6.3 \times 10^4$ | $9.8 \times 10^2$ | $9.4 \times 10^3$ | $6.4 \times 10^2$ |

[a]Responder cells were pooled from three mice in each group. Lungs were first minced and treated with colagenase. Plastic adherent cells were removed and twofold dilutions of responder cells were incubated for 5 days with PR8-infected, irradiated BALB/c splenocytes. Microcytotoxicity was carried out against PR8-infected or noninfected P815 cells and 1/pCTL frequency was estimated by linear regression ($-1.0 < r < -0.97$).
[b]Total pCTL number/organ was estimated taking into account the total number of separated cells.
[c]Mice were immunized with $3 \times 30$ $\mu g$ NPV1 at day 1, 3, and 6 after birth.

infectious challenge. Taken together, the data show that newborn mice immunized with a plasmid encoding NP of PR8 virus develop specific CTL immunity like the adult mice. This suggests that continuous exposure of neonate lymphocytes to antigens following DNA immunization leads to priming rather than tolerance induction. Furthermore, the boost effect of the PR8 virus noted in 4-week-old mice previously immunized with NPV1 as neonates indicated that significant CTL priming occurred during the first month of life. This is unexpected in light of previous studies that suggested that neonates are more susceptible to high-dose tolerance induction (Billingham et al., 1956). Thus, the expression of the antigen following DNA injection does not reach the threshold required for peripheral tolerance in the case of NPV1 plasmid. It results that the professional APCs are mainly involved in the presentation of antigen to the neonatal lymphocytes following NPV1 injection. As recently shown, although neonate lymphocytes were susceptible to tolerance induction when exposed to nonenriched populations of splenic APC, dendritic cells were able to prime a cytotoxic immune response in newborn mice (Ridge et al., 1996). Further, i.m. injection of NPV1 in newborn mice was not followed by central tolerance because 1- and 3-month-old mice responded to PR8 virus. This result, together with the observation that progenies of mothers immunized with NPV1 mounted normal CTL responses to the PR8 virus (manuscript in preparation), suggested that only very small amounts of NP protein are eventually released into the circulation in quantities below the threshold required for central tolerance induction.

CTLs primed by neonatal immunization with NPV1 displayed a similar specificity to those induced by adult immunization. This conclusion is supported by the presence of CTLs specific for the immunodominant NP 147-155 epitope previously characterized in adult mice (Taylor et al., 1987) and by the cross-reactivity against two virus strains of a distinct subtype. Thus, the development of CTL repertoire might follow a distinct pattern compared, for example, to the development of the B-cell repertoire. Previous studies demonstrated a delayed acquisition

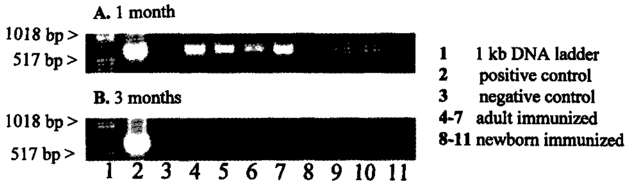

FIGURE 6 Persistence of NPV1 plasmid at the site of injection. PCR analysis of DNA extracted from tibial anterior muscles (adults, lanes 4 to 7) and gluteal muscles (neonates, lanes 8 to 11), 1 month (A) or 3 months (B) since the last inoculation with plasmid, was carried out as described in Materials and Methods. As a marker, we used 1-kb DNA ladder (lane 1) as a positive control NPV1 plasmid that gives a 711-bp PCR product (lane 2) and as a negative control, CP plasmid (lane 3). The results were confirmed by a second, independent experiment.

of the ability of B cells to respond to cross-reactive epitopes of influenza virus (Cancro et al., 1979).

Quantitative rather than qualitative differences in terms of CTL immunity were noted between mice immunized with NPV1 as neonates or adults, respectively. Estimation of pCTL frequency in spleens of mice immunized as adults or neonates with NPV1 1 month previously showed a lower expansion of the virus-specific population in the latter. This seems to be due simply to the lower number of T cells in young organisms, because the plasmid persisted at least 1 month in both age groups. Furthermore, 3-month-old mice immunized as neonates with NPV1 displayed an enhanced protection and increased numbers of virus-specific pCTLs in the spleen compared to 1-month-old mice (manuscript in preparation). These results suggest that the development of T-cell repertoire together with the persistence of peripheral antigen exposure leads to a continuous expansion of the primed, virus-specific CTLs in young mice immunized as neonates with NPV1. A slower expansion of the virus-specific CTL pool in the immunized neonates may explain the reduced protection of 1-month-old mice compared to 3-month-old mice and adult mice challenged with one $LD_{100}$ of PR8 or HK virus. In addition, the $LD_{100}$ infectious doses for 1-month-old mice are probably lower compared to those of adult mice.

CTL induced by NPV1 immunization of neonates displayed cross-reactivity against multiple type A strains of influenza virus and recognized the major cross-reactive NP 147-155 epitope. This is particularly important in the case of neonates, because of a high antigenic polymorphism of influenza virus strains faced by a limited ability of the immune system to respond to microbes in the early period of life. In spite of the cross-reactivity of CTLs induced by NPV1 immunization, a slightly reduced heterologous protection was demonstrated in mice immunized with NPV1 as adults or neonates. This may be due to a reduced magnitude of the cross-reactive immunity conferred by NP in our experimental system and probably to a faster replication rate of the HK virus in the lungs. The discrepancy with a previous study, which showed complete heterologous protection (Ulmer et al., 1993), may be explained by differences in the method of infection. It is known that aerosol challenge leads to an extensive infection of the upper and lower respiratory tract that can lead to fatal DTH lesions of the lungs (Sullivan et al., 1976) even in the case of successful virus clearance. Thus, our results showed that even if most of the mice immunized with NPV1 cleared the pulmonary virus by day 7 following lethal homologous or heterologous challenge, fewer of them survived and completely recovered. Effective early recruitment of the virus-specific CTL at the site of infection leads to rapid limitation of infection, prompt decrease of pulmonary virus titer, and reduced DTH lesions. Although a slower recruitment of specific CTLs may be still followed by a reduction of virus titer, DTH lesions tend to be more extensive and may cause the death of the animal.

PCR analysis of DNA extracted from muscles at the site of injection showed a reduced persistence of NPV1 plasmid in mice injected as newborns. This could be due to either a faster degradation or to the lytic activity of CTLs toward somatic cells transfected with NPV1. The latter hypothesis seems less probable because the efficiency of CTL priming was higher in adult mice that displayed longer persistency of the plasmid. Instead, in the case of newborns injected with DNA in the gluteal muscle, the limited persistency might be caused by a higher diffusion of the plasmid into the rapidly proliferating tissues or to the increased degradation. In contrast, the anterior tibial muscles of adult mice rich in conjunctive tissue and largely composed of nondividing myocytes may facilitate the retention of the plasmid over longer intervals. It is not clear yet what is the relationship between the persistency of the immunological memory, the plasmid at the site of injection, and the antigenic stimulation. Whereas some studies showed that the duration of the memory is strictly dependent on the continuous exposure to antigens (Gray and Matzinger, 1991; Oehen et al., 1992), other studies indicated that memory cells represent a distinctive subset differentiated from naive cells following antigen priming (Akbar et al., 1988; Camp et al., 1991).

DNA-based immunization presents some advantages over classical vaccine approaches currently employed in neonates and infants. Whereas inactivated and subunit vaccines prime mostly a humoral immune response, neonatal DNA vaccination with plasmids encoding NP and HA of influenza virus is followed by synergistic cellular and humoral immunity (manuscript in preparation). Other strategies like the use of live-attenuated vectors (Aggarwal et al., 1990; Tartaglia et al., 1992) or strong adjuvants (Harding et al., 1991) lead to generation of CTL immunity, but side effects and cost-related problems may eventually limit their large-scale use. In the case of live-attenuated vaccines, the strong tendency of neonates to develop a Th2 type of immune response to some viral epitopes may explain the limited efficacy of CTL induction (Barrios et al., 1996). The prolonged stimulation of the immune system following DNA injection may circumvent the requirement for boosts carried out in the case of inactivated vaccines and may lead to a long-term immunity. Regarding the possible side effects like oncogenesis, a recent study estimated that the rate of mutagenesis induced by DNA injection is significantly lower than the spontaneous chance of genome mutation (Nichols et al., 1995). In light of these properties, large-scale DNA vaccination strategies may be used to prevent the horizontal or vertical transmission of serious infectious diseases in infants and children, caused by HIV, HBV, Plasmodium, and so forth, in endemic geographical regions. Conversely, newborn DNA vaccination may be employed to a limited extent during periods of epidemics or in targeted recipients, for prevention of diseases transmitted by filoviruses, influenza virus, RSV, rotaviruses, and other infectious agents.

In conclusion, these results show that newborn DNA vaccination with a plasmid-encoding NP of influenza virus primed a CTL response, like in adult organisms. The cellular immunity primed by NPV1 injection of neonates was cross-reactive against various type A strains of influenza virus and mediated a protective effect in terms of both survival and virus clearance. The protectivity conferred by NPV1 immunization was associated with early local recruitment of specific pCTLs following aerosol infection with influenza virus. It results that continuous exposure of the neonate immune system to antigens following DNA-based immunization leads to priming, rather than tolerance induction as predicted from the higher susceptibility of newborns to high-zone tolerance. Based on these findings, DNA immunization may be used in infants and children to prevent the transmission of serious infectious diseases.

### MATERIALS AND METHODS

### Mice

BALB/c mice (H-$2^d$) were purchased from Jackson Laboratory (Bar Harbor, ME). Mice were housed and bred at Mount Sinai Animal Care Facility according to federal and local regulations.

### Plasmids, Peptide, and Viruses

NPV1 plasmid was constructed at Merck Research Laboratories (West Point, PA) by inserting the open reading frame of the NP gene of A/PR8/34 virus into the Bgl II site of a mutated pBR322 plasmid, containing 1.96 kb of enhancer, promoter, and intron A of the initial early gene of CMV (Ulmer et al., 1993). The control plasmid (CP) was obtained by excising the NP open reading frame from NPV1 plasmid. The plasmids were propagated in *E. Coli* and purified by the alkaline lysis method. DNA was ethanol-precipitated and resuspended in 0.9% saline to a concentration of 30 $\mu$g/100 $\mu$l for immunization.

The synthetic peptide NP 147-155 with the sequence TYQRTRALV, which is a major CTL epitope in the H-$2^d$ haplotype (Taylor et al., 1987), was synthesized in the Protein Core Facility of Mount Sinai School of Medicine. The NP 147-155 peptide was used for coating P815 mastocytoma target cells at a concentration of 10 $\mu$g/2.5 $\times$ $10^4$ target cells/ml.

The influenza virus strains A/PR8/34 (H1N1), A/HK/68 (H3N2), A/Japan/57 (H2N2), and B/Lee/40 were grown in an allantoic cavity of 10-day-old embrionated hen eggs. Allantoic fluids were harvested 48 hr later and stored at $-80$°C.

### Immunization and Infection

Adult BALB/c mice were immunized with $3 \times 30\ \mu g$ NPV1 or CP plasmid at 3-week intervals in the anterior tibial muscle of the right leg. Neonates were immunized with $3 \times 30\ \mu g$ of plasmid at day 1, 3, and 6 after birth in the right gluteal muscle. Some of mice were immunized i.p. with live PR8 virus diluted in 200-$\mu$l saline at a dose of $1 \times 10^4$ $TCID_{50}$.

Mice were challenged with aerosols containing a lethal dose ($LD_{100}$) of $1.5 \times 10^4$ $TCID_{50}$ PR8 or $1.5 \times 10^5$ $TCID_{50}$ HK virus. The allantoic fluid was diluted in saline and the infection was carried out using an aerosol chamber to which a nebulizer (Ace Glass, NY) was attached. Immunized and control mice were infected simultaneously. Mice were observed each day after infection in terms of survival and weight. Long-term survivors were considered the mice that recovered their original weight and did not display any infectious virus in their lungs at day 20 after challenge.

### Estimation of Pulmonary Virus Titer

Three mice in each group were sacrificed by cervical dislocation at day 3 and 7 after challenge and the lungs were harvested and homogenized in PBS-0.1% gelatin. Triplicates of tenfold dilutions from lung homogenates were prepared in DMEM-1% BSA and incubated for 1 hr with MDCK (Madine Darby Kidney Carcinoma) cells previously washed with Trypsin-EDTA medium. After adding DMEM-10% FCS, the plates were incubated for 48 hrs at 37°C and 5% $CO_2$. Supernatants were harvested and co-incubated for 30 min at room temperature with a suspension of 0.5% chicken red blood cells in 96-well round-bottom flexible plates (Falcon) as previously described (Isobe et al., 1994). Virus titers were estimated by interpolation of the dilution that showed hemagglutination in 50% of wells (Reed and Muench, 1938).

### Cell Preparation and Cytotoxic Assay

Spleens and lungs were aseptically removed from immunized or infected mice and single-cell suspensions were prepared by mincing and passing the tissue through cell strainers (Falcon). The lung tissue was pretreated with 8 U/ml collagenase (Sigma, MO) for 90 min at 37°C, as described previously (Stein-Streilein et al., 1983). The plastic adherent cells were removed and erythrocytes were lysed by hypotonic shock. Primary cytotoxic assay was carried out by incubating various numbers of effector cells immediately separated from organs, with $5 \times 10^3$ $^{51}Cr$-labeled P815 target cells in presence of NP147-155 peptide or previously infected with PR8 virus ($1 \times 10^4$ $TCID_{50}/1 \times 10^6$ target cells for 1 hr at 37°C). After 5-hr incubation in U-bottom plates at 37°C in 5% $CO_2$, the supernatants were harvested and radioactivity released was determined in a $\gamma$-counter (Automatic/Wallac-Finland).

For the secondary cytotoxic assays, mixed lymphocyte cultures were prepared and incubated for 5 days in RPMI supplemented with FCS 10%, 50 $\mu$M 2-mercaptoethanol, 1% nonessential amino acids, and 2% HEPES buffer, at a density of $2 \times 10^6$ responder cells/ml. Equal numbers of irradiated, PR8-infected, haplotype-compatible splenocytes ($10^8$ cells in 1.5 ml DMEM-1% BSA + $2 \times 10^5$ $TCID_{50}$ virus at 37°C for 1 hr) were added to cultures. The secondary cytotoxic assay was conducted as described previously, after carefully washing the effector cells obtained from the cultures. Background activity was estimated using noncoated, noninfected P815 cells as targets. All results were expressed as % specific lysis = [100 × (actual-spontaneous release)/(maximum-spontaneous release)] − background release ±SD of triplicate determinations.

### pCTL Frequency Estimation by Limiting Dilution Analysis

To evaluate the frequency of pCTL in spleens and lungs of immunized and infected mice, single-cell suspensions were prepared and incubated as twofold dilutions (six dilution steps, beginning with $2.5 \times 10^5$ cells/well) and 24 replicates for each dilution, in 96-well round-bottom plates. Before 5-day incubation at 37°C and 5% $CO_2$, $2.5 \times 10^5$ irradiated, PR8-infected, haplotype-matched splenocytes were added to each well. Individual cultures were tested against PR8-infected or noninfected $^{51}Cr$-labeled P815 target cells. Positive wells were considered those that displayed a percentage release greater than background + 3 × SD. The percentage of cultures in every dilution step regarded as negative for specific cytotoxicity was plotted logarithmically against the number of responder cells/well and the 1/pCTL frequency was estimated after linear regression by the intercept at 37% negative cultures (Lefkovits and Walkmann, 1979). All linear regression coefficients $r$ were between $-1$ and $-0.95$.

### Estimation of IFN$\gamma$ Production by T Cells

A number of $2 \times 10^5$ T cells purified on nylon-wool columns (Nycomed Pharma, Norway) were incubated with $1 \times 10^5$ irradiated BALB/c splenocytes in the presence of various concentrations of NP 147-155 peptide in 96-well flat-bottom plates for 4 days. Recombinant mouse IL-2 (Boehringer Mannheim) at a concentration of 6 U/ml was added to the cultures. Supernatants were harvested and IFN$\gamma$ or IL-4 concentration was estimated by ELISA (BioSource, Camarillo, CA). In alternative protocols, $CD4^+$ T cells were previously depleted using magnetic beads coupled with goat anti-rat IgG (PerSeptive Diagnostics, Cambridge, MA) and rat anti-CD4 mAb (GK 1.5).

### Plasmid Detection by PCR

Injected and noninjected muscle tissues were removed 1 or 3 months after completion of immunization with NPV1 plasmid, immediately frozen in ethanol-dry ice and stored at −80°C. Frozen tissue was thawed and homogenized in lysis buffer (25 mM Tris, 2 mM CDTA, 2 mM DTT, 10% glycerol, and 1% Triton X), as previously described (Ulmer et al., 1993). After phenol-chloroform extraction of DNA, 40-cycle PCR reactions were carried out using a PCR Reagent System Kit (Life Technologies) with the following NP-specific primers:

forward: 5′-CATTGTCTAGAATTT-GAACTCCTCTAGTGG

reverse: 5′-GGCCGTCGACCATGAT-GATCTGGCATTCC

PCR products of 711 bp were visualized using ethidium-bromide-stained agarose gels.

### Statistical Analysis

In the case of normal distributions like $\log_{10}$ of pulmonary virus titers or cytokine concentrations, we applied Student's $t$ test after running a statistical test for the sample variances ($F$ test). In the case of binomial distributions like the survival data, we computed the $p$ values of statistical significance by Fisher's exact test (Rosner, 1995).

### *Acknowledgements*

We thank Dr. Margaret Ann Liu (Merck Research Laboratories, West Point, PA) for the NPV1 plasmid, Scott Kerns (Mount Sinai School of Medicine, New York) for his technical assistance, and Dr. Peter Palese (Mount Sinai School of Medicine) for his helpful advice. This work was in part supported by Grant R01AI37115 from NIAID, NIH, and in part by a contract from Alliance Pharmaceutical, San Diego, CA.

### *References*

Adkins B., Ghanei A. and Hamilton K. (1993) Developmental regulation of IL-4, IL-2 and IFN-$\gamma$ production by murine peripheral T lymphocytes. *J. Immunol.* **151**: 6617-6626.

Aggarwal A., Kumar S., Jaffe R., Hone D., Gross M. and Sadoff J. (1990) Oral Salmonella: Malaria circumsporozoite recombinants induce specific CD8 cytotoxic T cells. *J. Exp. Med.* **172**: 1083-1090.

Akbar A.N., Terry L., Timms A., Beverley P.C.L. and Janossy G. (1988) Loss of CD45R and gain of UCHL1 reactivity is a feature of primed T cells. *J. Immunol.* **140**: 2171-2178.

Allsopp C.E., Plebanski M., Gilbert S., Sinden R.E., Harris S., Frankel G., Dougan G., Hioe C., Nixon D., Paoletti E., Layton G. and Hill A.V. (1996) Comparison of numerous delivery systems for the induction of cytotoxic T lymphocytes by immunization. *Eur. J. Immunol.* **26**: 1951-1959.

Barrios C., Brawand P., Berney M., Brandt C., Lambert P.-H. and Siegrist C.-A. (1996) Neonatal and early life immune responses to various forms of vaccine antigens qualitatively differ from adult responses: Predominance of a Th2 biased pattern which persists after adult boosting. *Eur. J. Immunol.* **26**: 1494-1496.

Billingham R., Brent L. and Medawar P. (1956) Quantitative studies on tissue transplantation immunity. III. Actively acquired tolerance. *Proc. Roy. Soc. London* **239**: 44-45.

Bona C., Lieberman R., Chien C.C., Mond J., House S., Green I. and Paul W.E. (1978) Immune response to levan. I. Kinetics and ontogeny of anti-levan and anti-inulin antibody response and expression of cross-reactive idiotype. *J. Immunol.* **120**: 1436-1442.

Camp R.L., Kraus T.A., Birkeland M.R. and Pure E. (1991) High levels of CD44 expression distinguish virgin from antigen-primed B cells. *J. Exp. Med.* **173**: 763-766.

Cancro M.P., Wylie D.E., Gerhard W. and Klinman N.R. (1979) Patterned acquisition of the antibody repertoire: Diversity of the hemagglutinin specific B cell repertoire in neonatal BALB/c mice. *Proc. Natl. Acad. Sci. USA* **76**: 6577-6581.

Davis H.L., Michel M.-L. and Whalen R.G. (1993) DNA-based immunization induces continuous secretion of hepatitis B surface antigen and high levels of circulating antibody. *Human Molecular Genetics* **2**: 1847-1851.

Epstein S.L., Misplon J.A., Lawson C.M., Subbarao E.K., Connors M. and Murphy B.R. (1993) β2-Microglobulin deficient mice can be protected against Influenza A infection by vaccination with Vaccinia-Influenza recombinants expressing Hemagglutinin and Neuraminidase. *J. Immunol.* **150**: 5484-5493.

Forsthuber T., Yip H.C. and Lehmann P.V. (1996) Induction of Th1 and Th2 immunity in neonatal mice. *Science* **271**: 1728-1731.

Gray D. and Matzinger P. (1991) T cell memory is short-lived in the absence of antigen. *J. Exp. Med.* **174**: 969-974.

Griffiths J.C., Berrie E.L., Holdsworth L.N., Moore J.P., Harris S., Senior J.M., Kingsman S.M. and Adams S.E. (1991) Induction of high-titer neutralizing antibodies using hybrid human immunodeficiency virus V3-Ty viruslike particles in a clinically relevant adjuvant. *J. Virol.* **65**: 450-456.

Harding C.V., Collins D.S., Kanagawa O. and Unanue E.R. (1991) Liposome-encapsulated antigens engender lysosomal processing for class II MHC presentation and cytosolic processing for class I presentation. *J. Immunol.* **147**: 2860-2863.

Isobe H., Alt F., Bona C.A. and Schulman J. (1994) Intact anti-influenza virus immune response in targeted k-deficient mice. *Viral Immunol.* **7**: 25-30.

Krug R.M. (1989) The Influenza Viruses (New York: Plenum Press).

Lefkovits I. and Walkmann H. (1979) Limiting Dilution Analysis of Cells in the Immune System (Cambridge: Cambridge University Press).

Lin Y.-L. and Askonas B. (1981) Biological properties of an Influenza A virus-specific killer T cell clone. Inhibition of virus replication in vivo and induction of delayed-type hypersensitivity reactions. *J. Exp. Med.* **154**: 225-234.

Lukacher A.E., Braciale V.L. and Braciale T.J. (1984) In vivo effector function of influenza virus specific cytotoxic T lymphocyte clones is highly specific. *J. Exp. Med.* **160**: 814-826.

Nair S., Zhou F., Reddy R., Huang L. and Rouse B. (1992) Soluble proteins delivered to dendritic cells via pH-sensitive liposomes induce primary cytotoxic T lymphocyte responses in vitro. *J. Exp. Med.* **175**: 609-612.

Nichols W.W., Ledwith B.J., Manam S.V. and Troilo P.J. (1995) Potential DNA vaccine integration into host cell genome. *Ann. N.Y. Acad. Sci.* **772**: 30-39.

Oehen S., Waldner H., Kundig T.M., Hengartner H. and Zinkernagel R.M. (1992) Antivirally protective cytotoxic T cells memory to lymphocytic choriomeningitis virus is governed by persisting antigen. *J. Exp. Med.* **176**: 1273-1281.

Reed L.J. and Muench H. (1938) A simple method of estimating fifty per cent endpoints. *Amm. J. Hyg.* **27**: 493-497.

Ridge J.P., Fuchs E.J. and Matzinger P. (1996) Neonatal tolerance revisited: Turning on newborn T cells with dendritic cells. *Science* **271**: 1723-1726.

Robinson H.L., Hunt L.A. and Webster R.G. (1993) Protection against a lethal influenza virus challenge by immunization with a hemagglutinin expressing plasmid DNA. *Vaccine* **11**: 957-960.

Rosner B.A. (1995) Fundamentals of Biostatistics, 4th ed. (City: Duxbury Press).

Sarzotti M., Robbins D.S. and Hoffman P.M. (1996) Induction of protective CTL responses in newborn mice by a murine retrovirus. *Science* **271**: 1726-1728.

Stein-Streilein J., Bennet M., Mann D. and Kumar V. (1983) Natural killer cells in mouse lung: Surface phenotype, target preference and response to local influenza virus infection. *J. Immunol.* **131**: 2699-2704.

Sullivan J.L., Mayner R.E., Barry D.W. and Ennis F.A. (1976) Influenza virus infection in nude mice. *J. Infect. Dis.* **133**: 91-94.

Tartaglia J., Perkus M.E., Taylor J., Norton E.K., Audonet J.-C., Cox W.I., Davis S.W., Van der Hoeven J., Meignier B., Riviere M., Languet B. and Paoletti E. (1992) NYVAC: A highly attenuated strain of vaccinia virus. *Virology* **188**: 217-232.

Taylor P.M., Davey J., Howland K., Rothbard J.B. and Askonas B.A. (1987) Class I MHC molecules rather than other mouse genes dictate influenza epitope recognition by cytotoxic cells. *Immunogenetics* **26**: 267-272.

Townsend A., Gotch F.M. and Davey J. (1985) Cytotoxic T cells recognize fragments of the influenza nucleoprotein. *Cell* **42**: 457-467.

Ulmer J.B., Donnely J.J., Parker S.E., Rhodes G.H., Felgner P.L., Dwarki V.J., Gromkowski S.H., Randall Reck R., DeWitt C.M., Friedman A., Hawe L.A., Leander K.R., Martinez D., Perry H.C., Shiver J.W., Montgomery D.L. and Liu M.A. (1993) Heterologous protection against influenza virus injection of DNA encoding a viral protein. *Science* **259**: 1745-1749.

Virelizier J.-L. (1975) Host defenses against influenza virus: The role of anti-hemagglutinin antibody. *J. Immunol.* **115**: 434-439.

Whalen R.G. (1996) DNA vaccines for emerging infectious diseases: What if? *Emerg. Infect. Dis.* **2**: 168-175.

Yankauckas M.A., Morrow J.E., Parker S.E., Abai A., Rhodes G.H., Dwarki V.J. and Gromkowski S.H. (1993) Long-term anti-nucleoprotein cellular and humoral immunity is induced by intramuscular injection of plasmid DNA containing NP gene. *DNA Cell Biol.* **12**: 771-776.

Yap K.L., Ada G.L. and McKenzie I.F.C. (1978) Transfer of specific cytotoxic T lymphocytes protects mice inoculated with influenza virus. *Nature* **273**: 238-239.

